# An adaptive planning strategy in carbon ion therapy of pancreatic cancer involving beam angle selection

**DOI:** 10.1016/j.phro.2022.01.005

**Published:** 2022-02-12

**Authors:** Motohiro Kawashima, Mutsumi Tashiro, Maria Varnava, Shintaro Shiba, Toshiaki Matsui, Shohei Okazaki, Yang Li, Shuichiro Komatsu, Hidemasa Kawamura, Masahiko Okamoto, Tatsuya Ohno

**Affiliations:** a Gunma University Heavy Ion Medical Center, 3-39-22 Showa-Machi, Maebashi, Gunma, Japan; b Department of Radiation Oncology, Gunma University Graduate School of Medicine, 3-39-22 Showa-Machi, Maebashi, Gunma, Japan

**Keywords:** Adaptive therapy, Carbon-ion therapy, Pancreatic cancer, Radiotherapy

## Abstract

**Background and purpose:**

In carbon-ion radiotherapy for pancreatic cancer, altered dose distributions due to changes in the gastrointestinal gas volume and anatomy during irradiation are an unresolved therapeutic issue. We developed and investigated an adaptive strategy involving beam angle selection to improve dose distributions in pancreatic cancer.

**Materials and methods:**

In the adaptive strategy, multiple beams were prepared with angles similar to those of the conventional strategy, and the beam that best reproduces the dose distribution of the treatment plan was used. The dose distributions of the adaptive strategy were compared with those of the conventional strategy for five patients. Patients underwent computed tomography (CT) before every irradiation. The adaptive strategy was evaluated using the same irradiation schedule as that of the conventional method and an adjusted method based on anatomical changes per fraction. Dose distributions on the pre-treatment CT and accumulated dose distributions on the treatment planning CT were evaluated using the volume receiving ≥95% of the prescription dose (V95) from the clinical target volume (CTV) between strategies.

**Results:**

There were significant differences in the CTV V95 values for the pre-treatment CT between all strategies. The median (range) CTV V95 for the conventional strategy was 92.7% (87.1–96.1%), for the proposed adaptive strategy without adjusted schedules was 96.9% (95.1–97.8%), and for the proposed strategy with adjusted schedules was 97.8% (96.5–99.2%).

**Conclusions:**

The adaptive strategy can improve target coverage for the pre-treatment CT and accumulated dose distributions for the treatment planning CT without increasing the dose to critical organs.

## Highlights

• In this study, we proposed a new adaptive strategy.
• A new option in treatment planning for particle therapy was considered.
• The high dose region for OARs was similar for the proposed and conventional strategies.
• The objective was to improve target coverage of the accumulated dose distribution.

## 1 Introduction

Pancreatic cancer is one of the most common causes of cancer-related death [1]. High-precision radiotherapy, such as intensity-modulated radiation therapy, stereotactic body radiation therapy, and particle therapy, allow the delivery of high therapeutic doses and offer favorable local control, facilitating a favorable clinical and survival course. Furthermore, several studies have shown their effectiveness in pancreatic cancer, with a positive relationship between increased dose and clinical outcomes [2], [3], [4]. Charged particle radiotherapy is characterized by a highly conformal dose distribution due to a sharp dose fall-off around the target volume [5], [6]. Nevertheless, reproducing the treatment plan with accuracy in pancreatic cancer radiotherapy is difficult because of anatomical changes due to organ filling and tissue shrinkage or expansion [7], [8]. Moreover, previous studies on the motion of pancreatic cancer [9], [10] have reported maximum movements of 9 mm based on monitoring data with ultrasound and 15 mm based on observations with four-dimensional magnetic resonance imaging. Anatomical changes, which affect dose distributions, are particularly important factors in particle therapy [11], [12], [13].

Various irradiation directions have been investigated for particle radiotherapy of pancreatic cancer to address this issue [14], [15], [16], [17], [18]. Dreher et al. compared dose distributions under various conditions using 1–3 beams and reported that three-field configurations showed the best dose distributions [18]. Therefore, we assumed that the use of more beams results in better dose distributions. Furthermore, various robustness optimization methods have been developed recently [19], [20], [21], [22], [23]. Robustness was ensured by statistically processing the effects of setup errors and target deformation during treatment. Van Der Horst et al. reported that the amount of gastrointestinal gas can vary greatly [24]. Therefore, changes to the beam path due to gastrointestinal gas are expected to be large. Furthermore, the stomach and small intestine are in close proximity to the target in almost all cases. It is difficult to deal with this statistically when the beam is non-robust and the error is large.

We propose a novel adaptive treatment strategy to reduce the impact of anatomical changes. In this study, we confirmed the effectiveness of the proposed strategy by comparing dose distributions.

## 2 Material and methods

### 2.1 Patient data

The medical ethics committee of our medical faculty consented to this *in silico* study, and participants provided written informed consent. This research was conducted in accordance with the principles of the Declaration of Helsinki. Five patients with pancreatic cancer who underwent irradiation in 2019 were included in this study. Three out of these five patients had pancreatic head cancer, and the other two had pancreatic body cancer. All patients underwent computed tomography (CT) for treatment planning (plan-CT) and positioning before all irradiations (pre-CT). For CT imaging, a 3-mm-thick shell (Taisei Medical Co., Osaka, Japan) and a patient immobilization device (Mold Care; Alcare, Tokyo, Japan) were employed. The slice thickness was set to 2 mm. Plan-CTs were acquired 2 weeks before the first irradiation, and pre-CTs for positioning were acquired during the irradiation period.

Target delineation was performed as described by Shinoto et al. [25], [26]. The gross tumor volume (GTV) was defined using contrast-enhanced CT, magnetic resonance, and 18F-fluorodeoxyglucose positron emission tomography images. The clinical target volume (CTV) was defined as the GTV plus a 5-mm margin, including the prophylactic lymph node area around the pancreas. There were two types of planning target volumes (PTVs): PTV1 was defined as the CTV area plus a 2–5 mm margin depending on the target and gastrointestinal tract locations, and PTV2 was defined with a focus on GTV.

### 2.2 Treatment planning

Treatment plans were created using plan-CT images with the XiO-N system (ELEKTA, Stockholm, Kingdom of Sweden and Mitsubishi Electric, Tokyo, Japan) for each patient [27].

Our facility uses Gy (RBE) as the unit of the clinical dose, which was calculated based on the physical dose and relative biological effectiveness (RBE) [28]. At our hospital, a prescription dose of 55.2 Gy (RBE) is administered in 12 fractions (4.6 Gy (RBE) per fraction) to pancreatic cancer patients. Two different strategies were followed to create treatment plans; the conventional and proposed adaptive strategies. These strategies are described in Section 2.3 and Section 2.4. Dose distributions were calculated using a passive scattering method with beams from various angles. We adopted a gated irradiation method so that the respiratory motion was small. Therefore, we did not use an internal target volume. Beam angles were selected according to the guidelines of the International Electrotechnical Commission. This study was simulated under the assumption of a rotating gantry since rotating body position causes anatomical changes.

### 2.3 Conventional treatment strategy

The conventional treatment strategy refers to the standard strategy based on the protocol for determining the irradiation direction, number, and schedule of carbon-ion therapy for pancreatic cancer in our institution. According to the standard strategy, the PTV1 is irradiated with a dose of 41.4 Gy (RBE) in nine fractions in the supine position (three times from three directions). The PTV2 is irradiated with a dose of 13.8 Gy (RBE) in three fractions in the prone position. For the purpose of this study, 10 patterns of CT images in the supine position (one plan-CT and nine pre-CTs) were employed because the dose distributions were assessed only for the PTV1, in which case the organs-at-risk (OARs) were near the target.

**Fig. 1:**
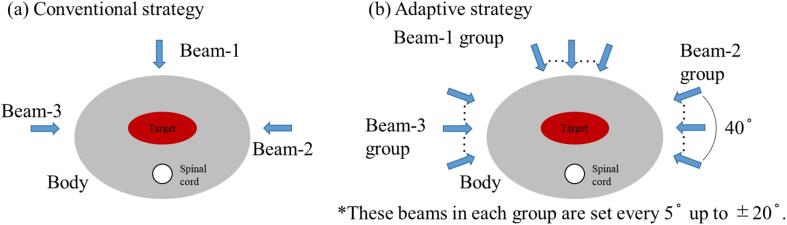
Schematics of treatment plans for both strategies. The schematics of treatment plan in the conventional strategy are shown in (a). The treatment plan has only three beams (beams 1, 2, and 3 at angles of 0°, 90°, and 270°, respectively). The schematics of treatment plan for the adaptive strategy are shown in (b). This treatment plan has three groups. Each beam group consists of nine directions in 5° increments up to ±20°.

In the treatment plans for the conventional strategy, the PTV1 was irradiated using three beams (beams 1, 2, and 3 at corresponding angles of 0°, 90°, and 270°, respectively). Each beam was used three times. The irradiation schedule included nine fractions (beams 1, 2, 3, 1, 2, 3, 1, 2, and 3). The treatment plan schematic and irradiation schedule of the conventional strategy are shown in Fig. 1(a) and Table 1, respectively.

**Table 1:** Irradiation schedule. The irradiation schedules in the conventional and adaptive strategies are fixed. In contrast, the irradiation schedules in the adaptive strategy with an adjusted schedule are determined so that the clinical target volume V95 is the highest. Therefore, any beam group is appropriate to use. All irradiation schedules include three irradiations from each direction.

Times	Conventional			Adaptive			Adaptive with an adjusted schedule		
	Beam-1	Beam-2	Beam-3	Beam-1 group	Beam-2 group	Beam-3 group	Beam-1 group	Beam-2 group	Beam-3 group
1st	○			○			The irradiation beam is determined so that the V95 of the CTV is the highest. Therefore, any group is fine		
2nd		○			○		*A constraint of the irradiation schedule was that each group was selected three times.		
3rd			○			○			
4th	○			○					
5th		○			○				
6th			○			○			
7th	○			○					
8th		○			○				
9th			○			○			

Total	3	3	3	3	3	3	3	3	3

### 2.4 Adaptive treatment strategy

The proposed adaptive treatment strategy is based on the concept of increasing the flexibility of treatment planning to improve dose distributions in pancreatic cancer. A single beam in particle radiotherapy can produce a flat dose distribution to a target. Furthermore, it is possible to irradiate the target with a single beam per day. Therefore, each beam can be considered as an independent treatment plan, making the repeated irradiation of the same beam unnecessary. We propose the addition of beam selection as a new degree of freedom to treatment planning.

The adaptive strategy includes the preparation of several beams with similar concepts in various directions and the selection of the beam with the least change in the dose distribution of the treatment plan. In this study, we prepared 3-beam groups corresponding to the beams used in the conventional strategy as shown in Fig. 1(b). Each beam group comprised beams in nine directions. The beam angles were set at −20° to +20° in 5° intervals from the beam direction of the conventional treatment plan. All beams from the same beam group had the same characteristics as those of the corresponding beam from the conventional treatment plan, such as target coverage and OAR dose. The beam with the highest CTV coverage in every group was selected. The CTV coverage of each beam was obtained from the dose distributions calculated on the pre-CT. For the irradiation schedule of the adaptive strategy, a beam group was selected instead of a beam.

The adaptive strategy has two patterns: the same irradiation schedule as that of the conventional strategy and an adjusted irradiation schedule. The adjusted irradiation schedule was designed so that the percentage of the CTV volume receiving ≥95% of the prescribed dose (CTV V95) had the highest value after the delivery of all nine fractions. At every fraction, the beam with the highest CTV V95 value was chosen. However, a constraint was set such that each beam group could be selected only three times. For each patient, the combination of nine beams (three beams from each group) that resulted to the highest possible CTV V95 was selected. The irradiation schedule for the adaptive strategy is shown in Table 1.

### 2.5 Experimental design

We evaluated the adaptive strategy with reference to the conventional strategy. The dose distributions on plan-CTs and pre-CTs with conventional and adaptive strategies were evaluated.

In addition, we discussed the irradiation schedule. The conventional strategy has a fixed irradiation schedule. The adaptive strategy was evaluated using the same irradiation schedule as that of the conventional strategy and an adjusted schedule based on the anatomical changes per fraction. In short, we compared a conventional strategy with a typical irradiation schedule and the adaptive strategy with typical and adjusted irradiation schedules.

### 2.6 Evaluation of each strategy

Beam data for each treatment plan were used to calculate the dose distributions on each pre-CT. The beam isocenter on the pre-CT was determined from patient positioning based on tumor-matching between the plan-CT and each pre-CT image. Targets and OARs were delineated on all CT images for each patient by physicians. These contours were used for the dose-volume histogram (DVH) assessments on each pre-CT.

At first, we evaluated DVH parameters for each pre-CT. Dose distributions were calculated for all nine pre-CT sets using all beams in each treatment plan. The target coverages for each beam were compared using DVH parameters, such as the CTV V95. In total, for each parameter there were 45 values (5 patients × 9 pre-CTs). Second, the target coverages were evaluated for each pre-CT and beam according to every irradiation schedule. Because our data were dependent and not normal, the Friedman test was used to investigate for significant differences in the parameters between irradiation schedules. Then, the Wilcoxon signed-rank test with Bonferroni correction was performed to compare each strategy, generating effect estimates and 95% confidence intervals. Bonferroni correction was used to account for multiple testing. Finally, we evaluated accumulated dose distributions for the plan-CT. The accumulated dose distributions were created from all pre-CTs and plan-CTs using rigid registration based on tumor-matching in Maestro (MIM Software Inc., Cleveland, USA). Rigid registration was selected because deformable image registration accuracy may not be acceptable when image intensity significantly varies in the presence of gastrointestinal gas [29]. The dose distributions for each pre-CT were transferred to the plan-CT and integrated on the plan-CT images to obtain the accumulated dose distributions. The contouring of the plan-CT was used for the evaluation.

## 3 Results

The CTV V95 values of all pre-CTs for all beams used in the adaptive strategy are summarized in Table 2. Detailed data for each patient are given in Table S1 (Supplementary Materials). By changing the beam angle from that used in the conventional strategy (0°, 90°, and 270°), it was observed that the V95 value was increased for different angles, showing that target coverage can be improved. Moreover, the beams that pass through the small intestine (such as the beam-2 group) may not be stable. There was a larger range in the V95 values of the beam-2 group compared with the other two groups. The median (range) CTV coverage for all beams in the conventional strategy was 94.3% (62.3–99.9%). Detailed data for the conventional strategy are shown in Fig. S1 (Supplementary Materials).

**Table 2:** Median and range of the percentage volumes receiving ≥ 95% of the prescribed dose values for all beams in the adaptive strategy.

Beam-1 group									
	Beam angle								
	340°	345°	350°	355°	0°	5°	10°	15°	20°
median (%)	95.3	95.9	96.3	95.5	95.5	95.4	95.0	94.2	93.4
min (%)	81.5	81.8	83.1	84.3	86.0	82.1	81.3	80.2	79.1
max (%)	98.9	99.6	99.8	99.0	99.9	99.8	99.5	99.3	99.5

Beam-2 group									

	Beam angle								
	70°	75°	80°	85°	90°	95°	100°	105°	110°

median (%)	86.9	88.6	90.6	92.8	93.4	94.2	94.9	94.3	94.6
min (%)	68.0	66.7	62.1	61.6	62.3	60.5	61.3	66.1	68.8
max (%)	95.5	96.5	97.6	97.7	99.3	98.8	99.0	99.1	99.1

Beam-3 group									

	Beam angle								
	250°	255°	260°	265°	270°	275°	280°	285°	290°

median (%)	95.3	96.0	95.6	95.0	94.9	94.5	94.2	92.6	91.7
min (%)	89.7	89.1	89.2	87.8	86.2	86.8	81.7	77.2	78.9
max (%)	99.3	99.3	99.2	99.6	99.2	99.0	99.0	99.2	99.1

**Fig. 2:**
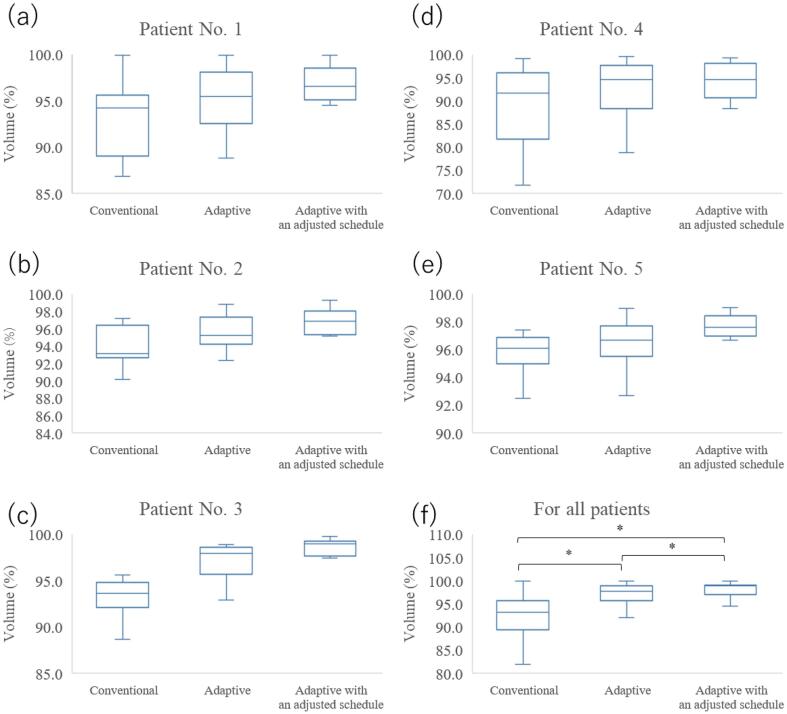
Box-plots of clinical target volume V95 values. The V95 values were calculated on all pre-CTs for each patient for all beams irradiated within each strategy. Patients No. 1–5 are shown in (a)-(e). In addition, (f) shows the V95 values of all patients for each strategy. There were significant differences between all strategies, with p-values less than 0.01. (*p < 0.01).

In Fig. 2(a)-(f), the CTV V95 values obtained from the beams and pre-CTs according to the irradiation schedule of each strategy are summarized as a box plot. The median and range of the V95 values in the conventional strategy for all patients were 92.7% and 87.1–96.1%, respectively. The corresponding V95 values were 96.9% and 95.1–97.8% for the adaptive strategy and 97.8% and 96.5–99.2% for the adaptive strategy with an adjusted schedule. The adaptive strategy with an adjusted schedule had the highest intermediate V95 value, whereas the conventional strategy had the lowest value. The CTV V95 values were significantly different between the conventional and adaptive strategies. Significant differences were observed between all strategies with p-values <0.01 (Fig. 2(f)). In the conventional strategy, only one patient (one of the most stable ones) had an intermediate CTV V95 value of >95% (patient No. 5). In contrast, in the adaptive strategy, all patients except patient No. 4 (one of the most variable ones) had an intermediate CTV V95 value of >95%.

**Fig. 3:**
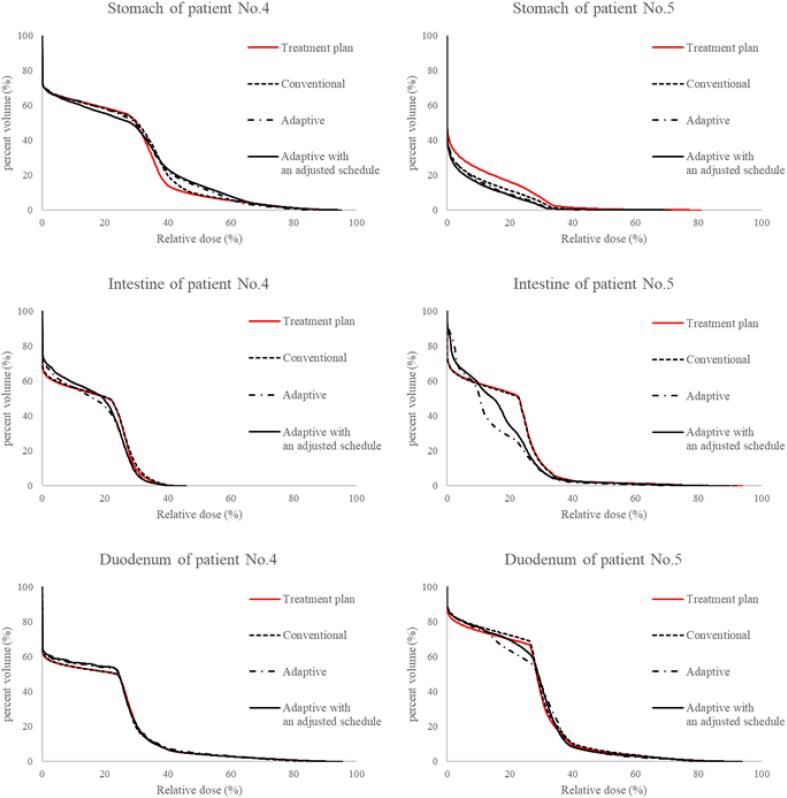
Dose-volume histograms (DVHs) obtained from the accumulated dose distributions for each treatment strategy. The left column shows the data of patient No. 4, and the right column shows the data of patient No. 5. From top to bottom: DVHs of the stomach, intestine, and duodenum.

Fig. 3 shows the DVHs calculated from the accumulated dose distributions for patients with the worst (patient No. 4) and best (patient No. 5) V95 values in the assessment with pre-CTs using the conventional strategy. The DVHs of the low dose region for OARs varied because of beam direction changes, although the DVHs of the high-dose areas for OARs were similar. Additional information about the OAR doses of the other patients and differences between the DVH parameters for all strategies is shown in Fig. S2 (Supplementary Materials). Data representing the difference between DVH parameters (V80, V60, and V40) for each OAR are summarized in Table S2 (Supplementary Materials).

**Fig. 4:**
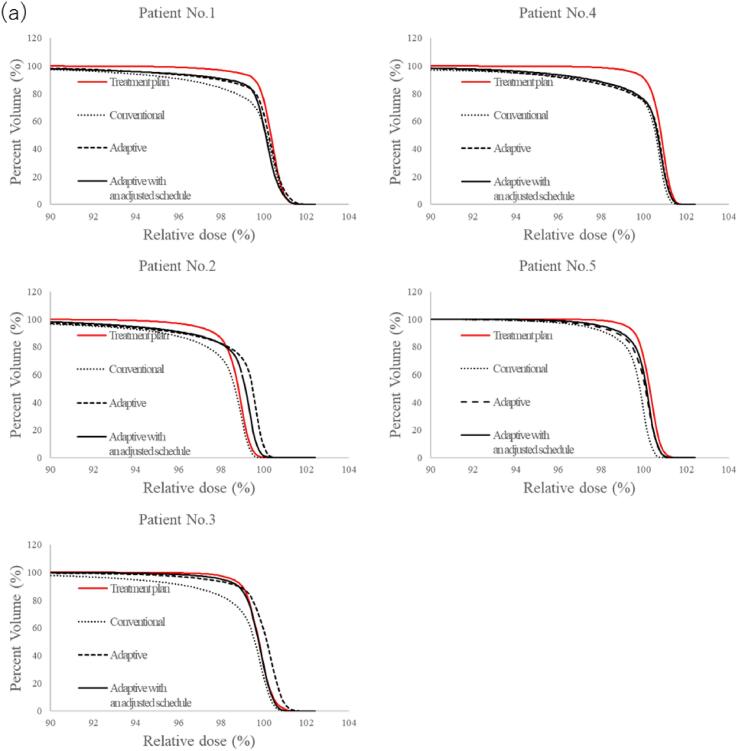
Dose-volume histograms (DVH) of the clinical target volume (CTV) obtained from the accumulated dose of each strategy for all patients. Here, “Treatment plan” corresponds to the DVH obtained from the treatment plan created based on the plan-CTs with the conventional strategy.

The CTV DVHs of each strategy for all patients, obtained from the accumulated dose distributions, are shown in Fig. 4. The accumulated dose distributions obtained from the adaptive strategies were improved compared with those of the conventional strategy. This can also be seen in Fig. S3 (Supplementary Materials), where accumulated dose distributions with each strategy are presented for patient 4. The adaptive strategies delivered high doses to the target, even to the area that received a low dose with the conventional strategy.

## 4 Discussion

We proposed an adaptive treatment strategy and showed its feasibility in mitigating the effect of anatomical changes during carbon-ion radiotherapy in pancreatic cancer. The CTV coverage for beams in the conventional strategy can be greatly reduced because of errors such as gastrointestinal gas change, CTV deformation, and setup errors. In contrast, the adaptive strategy was able to reduce the impact of errors. Therefore, our study demonstrated improved target coverage without the increased size of the high-dose area to OARs for the pancreatic cancer carbon-ion radiotherapy implemented with the adaptive strategy. Conversely, in the conventional strategy, dose calculation was performed according to the clinical procedure. However, when an unacceptable dose distribution is confirmed in clinical practice, the irradiation schedule may be changed, and the treatment plan can be adjusted.

The DVHs of the accumulated dose distributions implementing the adaptive strategy with and without adjusted schedules were compared. The adaptive strategy with adjusted schedules used the beam with the best CTV coverage in each pre-CT, considering CTV deformation and setup errors. There was no substantial difference in the DVHs of the accumulated dose distributions in the plan-CT without CTV deformation or setup errors. This suggests that correction for CTV deformation and setup errors could be expected even without an adjusted schedule if a sufficient compensatory PTV margin is provided.

Although the adaptive strategy in this study delineated the contours and calculated dose distributions for detailed analysis, the process is time-consuming and difficult to introduce in clinical practice. It may be necessary to improve the beam selection method by comparing the water equivalent path length, which influences dose distributions.

Furthermore, we discuss the advantages of this study compared with previous studies. First, another irradiation method with two dorsal fields aiming for the gap between the spinal cord and kidneys can be used [7]. This method was assumed as stable as the beam does not pass through the organs, such as the small intestine and stomach; thus, it would avoid the adjacent intestines and stomach only at the distal edge. Furthermore, a range calculation for particle therapy with a 3% error rate has been reported [30]. When the CTV and OARs are close, the PTV margin may be reduced. Moreover, Guy et al. reported an increase in respiratory motion based on the body position [31], and Fontana et al. reported that setup motion in the prone position was significantly greater than that in the supine position [32]. Therefore, irradiation with several beams in the supine position is considered a better irradiation method. Next, there have been many recent reports on adaptive therapy [33], [34], [35], showing that it can improve dose distributions. However, adaptive therapy can be time-consuming (>1 h duration), including re-contouring, plan optimization, and confirmation [35]. The optimization on the pre-CT may not result in an optimal treatment plan because of gastrointestinal gas movement over time [8]. In contrast, the adaptive strategy, wherein the beam is determined by beam range confirmation, uses a previously calculated irradiation beam. Hence, irradiation can be performed by rotating the gantry with a normal setup. The gantry speed is 2.5 min/rotation at the National Institute of Radiological Sciences.

This study had some limitations. Improvements regarding the study approach and investigated procedure should be addressed in future investigations. Although all data were compared to determine the irradiation schedule of the adaptive strategy, the same methodology cannot be implemented when there are no such data. It is necessary to change the beam selection method, such as performing comparisons within the beam range. However, the adaptive strategy alleviated the gastrointestinal gas problem by adding a new degree of freedom in treatment planning. Moreover, this novel strategy takes advantage of particle therapy characteristics, which are different from those of photon beams, and is expected to provide a new option for treatment planning in particle therapy. Furthermore, the adaptive strategy can improve accuracy for other sites, such as mucosal thickening in the head and neck, differently affected by angle.

In conclusion, the adaptive strategy proposed in this study effectively improves target coverage while maintaining similar OAR doses compared with those in conventional radiotherapy. This procedure can improve the clinical course of patients with pancreatic cancer by providing a new effective option and beam selection for particle therapy.

## Declaration of Competing Interest

The authors declare that they have no known competing financial interests or personal relationships that could have appeared to influence the work reported in this paper.
