# Supplementary material for: An adaptive planning strategy in carbon ion therapy of pancreatic cancer involving beam angle selection
Source: Phys Imaging Radiat Oncol. 2022 Feb 12;21:35–41. doi: 10.1016/j.phro.2022.01.005 (PMC8850338; doi:10.1016/j.phro.2022.01.005)
Supplement: Supplementary data 1 [file mmc1.docx]

**Supplementary materials**

The CTV V95 values of all pre-CTs for all beams used in the adaptive strategy are summarized for each patient in Table S1.

Table S1. Range (median) of the volumes receiving ≥95% of the prescribed dose values for each beam in the adaptive strategies.

| Beam-1 group | | | | | | | | | | |
| --- | --- | --- | --- | --- | --- | --- | --- | --- | --- | --- |
| Patient No. | | Beam angle | | | | | | | | |
|  | 340° | | 345° | 350° | 355° | 0° | 5° | 10° | 15° | 20° |
| 1 | **81.5-95.8 (92.1)** | | **81.8-97.0 (93.1)** | **83.1-98.0 (93.9)** | **84.3-99.0 (94.2)** | **88.3-99.9 (85.6)** | **82.1-99.8 (92.5)** | **81.3-99.4 (91.2)** | **80.2-99.3 (89.9)** | **79.1-99.0 (88.9)** |
| 2 | **91.1-96.8 (96.1)** | | **91.6-97.5 (95.9)** | **91.8-97.7 (96.0)** | **91.2-97.0 (96.1)** | **89.9-97.3 (96.1)** | **89.0-97.7 (96.8)** | **87.0-97.8 (96.4)** | **84.4-97.3 (94.6)** | **81.6-96.5 (91.8)** |
| 3 | **91.4-98.9 (94.5)** | | **93.6-99.6 (97.4)** | **94.8-99.8 (98.0)** | **92.8-99.0 (96.2)** | **89.0-97.6 (94.0)** | **89.8-98.2 (95.3)** | **90.5-98.4 (95.3)** | **90.1-98.3 (94.7)** | **89.8-98.2 (93.4)** |
| 4 | **92.5-98.4 (96.4)** | | **91.5-98.9 (96.5)** | **90.7-99.0 (96.0)** | **87.6-98.6 (93.9)** | **86.0-99.0 (92.6)** | **86.2-99.2 (93.1)** | **85.5-99.5 (92.7)** | **85.0-99.3 (92.9)** | **85.1-99.5 (93.2)** |
| 5 | **91.2-97.2 (96.0)** | | **91.5-97.6 (95.6)** | **92.0-98.2 (96.4)** | **92.7-98.7 (96.6)** | **92.5-99.0 (96.7)** | **92.0-98.6 (96.7)** | **90.9-98.2 (96.1)** | **91.0-98.1 (96.0)** | **90.8-98.0 (95.8)** |
| Beam-2 group | | | | | | | | | | |
| Patient No. | | Beam angle | | | | | | | | |
|  | 70° | | 75° | 80° | 85° | 90° | 95° | 100° | 105° | 110° |
| 1 | **77.4-94.2 (86.4)** | | **80.5-95.2 (88.6)** | **80.7-94.9 (87.7)** | **83.0-94.6 (88.6)** | **85.2-95.7 (90.2)** | **87.8-97.1 (92.5)** | **88.2-98.1 (93.2)** | **88.2-98.1 (93.8)** | **89.0-97.3 (94.5)** |
| 2 | **78.4-93.4 (91.0)** | | **84.7-95.7 (93.1)** | **90.6-96.9 (92.5)** | **90.7-97.1 (94.5)** | **90.2-99.3 (96.1)** | **90.6-98.8 (96.1)** | **91.0-99.0 (94.9)** | **93.2-99.1 (95.5)** | **93.3-99.0 (96.5)** |
| 3 | **68.0-86.9 (78.9)** | | **66.7-89.0 (83.6)** | **70.1-93.1 (89.8)** | **74.1-95.1 (92.7)** | **80.3-97.1 (94.3)** | **89.7-98.1 (96.3)** | **91.9-98.8 (98.0)** | **92.6-99.0 (98.5)** | **92.9-99.1 (98.4)** |
| 4 | **70.6-91.2 (85.1)** | | **68.4-89.9 (83.8)** | **62.1-86.0 (79.1)** | **61.6-87.3 (79.5)** | **62.3-88.9 (80.4)** | **60.5-88.4 (80.4)** | **61.3-87.6 (80.5)** | **66.1-90.8 (85.1)** | **68.8-92.5 (87.2)** |
| 5 | **91.3-95.5 (93.5)** | | **92.1-96.5 (94.4)** | **93.5-97.6 (95.9)** | **93.7-97.7 (96.2)** | **93.3-97.7 (96.1)** | **93.5-98.2 (96.6)** | **93.1-97.8 (95.9)** | **92.9-97.6 (94.7)** | **93.2-97.9 (94.4)** |
| Beam-3 group | | | | | | | | | | |
| Patient No. | | Beam angle | | | | | | | | |
|  | 250° | | 255° | 260° | 265° | 270° | 275° | 280° | 285° | 290° |
| 1 | **93.0-97.0 (94.7)** | | **92.0-95.5 (93.4)** | **91.7-94.7 (93.5)** | **90.8-95.0 (93.2)** | **89.3-94.9 (93.6)** | **89.1-94.5 (93.5)** | **88.8-94.8 (93.1)** | **86.7-935 (91.3)** | **85.4-92.6 (90.1)** |
| 2 | **89.7-96.4 (93.7)** | | **89.1-96.3 (93.5)** | **89.2-96.5 (93.4)** | **87.8-96.9 (93.1)** | **86.2-96.3 (92.5)** | **86.8-96.3 (91.6)** | **81.7-96.2 (91.8)** | **77.2-94.5 (88.1)** | **78.9-93.7 (86.5)** |
| 3 | **90.6-98.4 (94.8)** | | **91.9-99.0 (96.1)** | **92.2-99.0 (96.2)** | **92.6-99.6 (96.6)** | **91.7-99.2 (95.7)** | **91.5-99.0 (95.0)** | **86.5-98.7 (92.2)** | **82.2-98.6 (90.8)** | **79.1-97.1 (90.5)** |
| 4 | **90.3-98.0 (95.7)** | | **91.6-98.8 (96.4)** | **92.2-98.9 (96.7)** | **92.5-98.8 (96.3)** | **91.6-98.2 (96.0)** | **90.1-98.3 (96.1)** | **90.2-99.0 (96.2)** | **90.6-99.2 (97.1)** | **90.3-99.1 (97.0)** |
| 5 | **96.3-99.3 (98.3)** | | **96.5-99.3 (98.6)** | **96.5-99.2 (98.4)** | **96.1-99.2 (97.3)** | **95.6-99.0 (96.4)** | **95.2-98.2 (96.3)** | **94.3-97.3 (95.5)** | **94.8-97.4 (95.8)** | **95.6-97.4 (96.3)** |

*The unit for all data is %.

*Intermediate values >95%, between 90% and 95%, and <90% are shown in green, yellow, and red, respectively.

The volume receiving ≥95% of the prescription dose (V95) of the clinical target volume (CTV) for all pre-CTs obtained with the conventional strategy is summarized as a box-plot in Figure S1(a). These data are shown individually for each patient. The angles of beams 1, 2, and 3, shown in Figure S1(a), were 0°, 90°, and 270°, respectively. In addition, the CTV V95 values in pre-CTs for all patients are shown in Figure S1(b). These values consisted of 45 data (5 patients × 9 pre-CTs) that were evaluated considering the CTV on each pre-CT, and include all errors, such as gastrointestinal gas change, CTV deformation, and setup errors. The median values for beam 1, beam 2, and beam 3 were 95.5%, 93.4%, and 94.9%, with ranges of 86.0-99.9, 62.3-99.3, and 86.2-99.2, respectively. The Wilcoxon signed-rank test with Bonferroni correction was performed to compare the beams. The p-values for these tests were <0.001 (beam-1 and beam-2), 0.815 (beam-1 and beam-3), and 0.013 (beam-2 and beam-3). The dose distributions of beam-1 through the stomach and beam-3 through the liver were significantly more stable than those when beam-2 was used. Beam-2, which passed through the intestine, had the highest variation due to daily gastrointestinal gas volume changes.


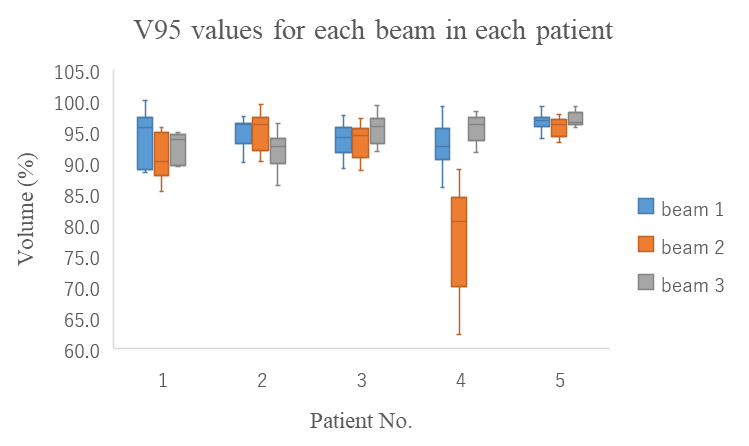
(a)


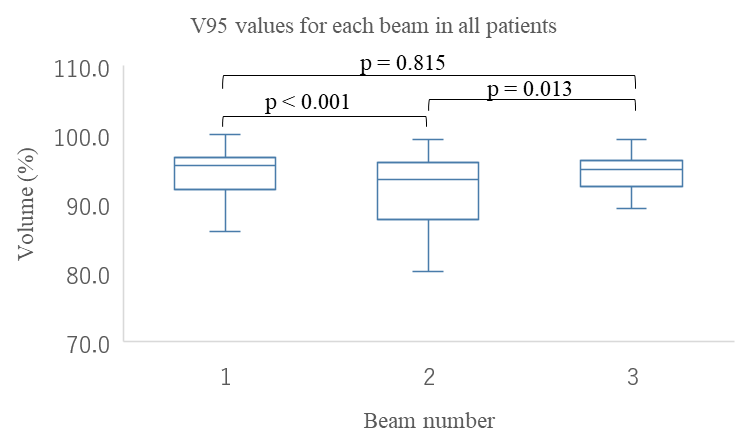
(b)

Figure S1. Box-plot of clinical target volume (CTV) V95 values.

(a) CTV V95 values on all pre-CTs. The V95 values were obtained from each beam in the conventional treatment plan. (b) Medians and ranges were calculated from the CTV V95 values. These V95 values were obtained from beams angled at the same direction on all pre-CTs for each patient.

Dose-volume histograms (DVHs) of organs-at-risk (OARs) determined from the accumulated dose distributions for each strategy are shown in Figure S2. The OARs include the duodenum, intestine, and stomach around the target. Figure S2 (a), (b), and (c) indicate results for patients No. 1, 2, and 3, respectively. Patient No. 3 underwent post-gastrectomy, and the stomach was not assessed. The difference between DVH parameters (V80, V60, and V40 for each OAR) are summarized in Table S2. DVH parameters (Vx) indicate the absolute volumes irradiated with ≥x% of the prescribed dose.

The largest V80 was in the small intestine for the adaptive treatment strategy for patient No. 3 at 4.98 cc. However, the average V80 for all patients was <2 cc for all strategies and OARs. In contrast, several values of >5 cc were found in V60 and V40 as shown in Table S2.


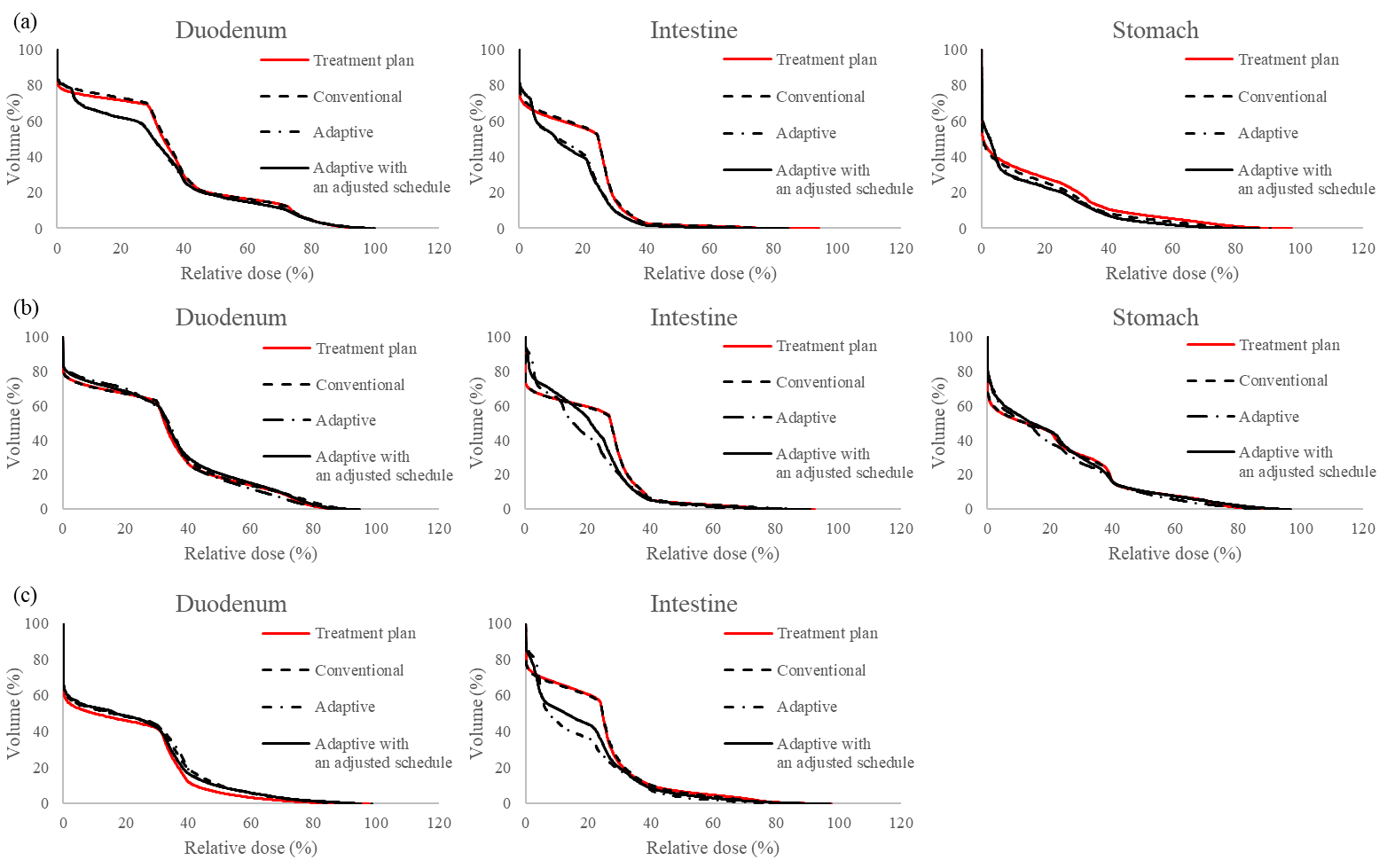
 Figure S2. Dose-volume histogram (DVH) of organs-at-risk calculated from the accumulated dose distributions for each strategy. Here, “Treatment plan” corresponds to the DVH obtained from the treatment plan created based on the plan-CTs with the conventional strategy.

1. DVHs for patient No. 1
2. DVHs for patient No. 2
3. DVHs for patient No. 3


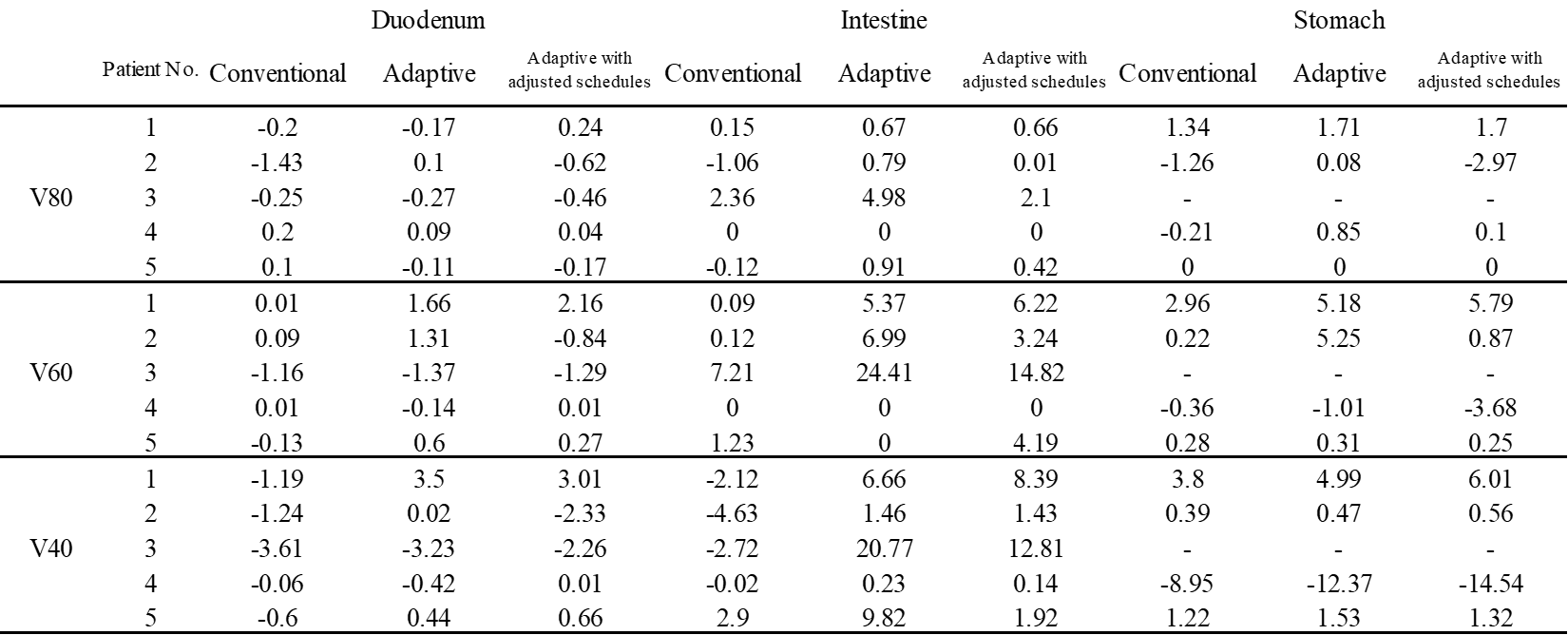
Table S2. Summary of the differences between dose-volume histogram (DVH) parameters (V80, V60, and V40 for each organs-at-risk). These differences were calculated by subtracting the DVH parameter of each strategy from the DVH parameter of the treatment plan. These values are expressed in cc.

This can also be seen in Figure S3, where accumulated dose distributions with each strategy are presented for patient 4. The adaptive strategies delivered high doses to the target, even to the area that received a low dose with the conventional strategy.


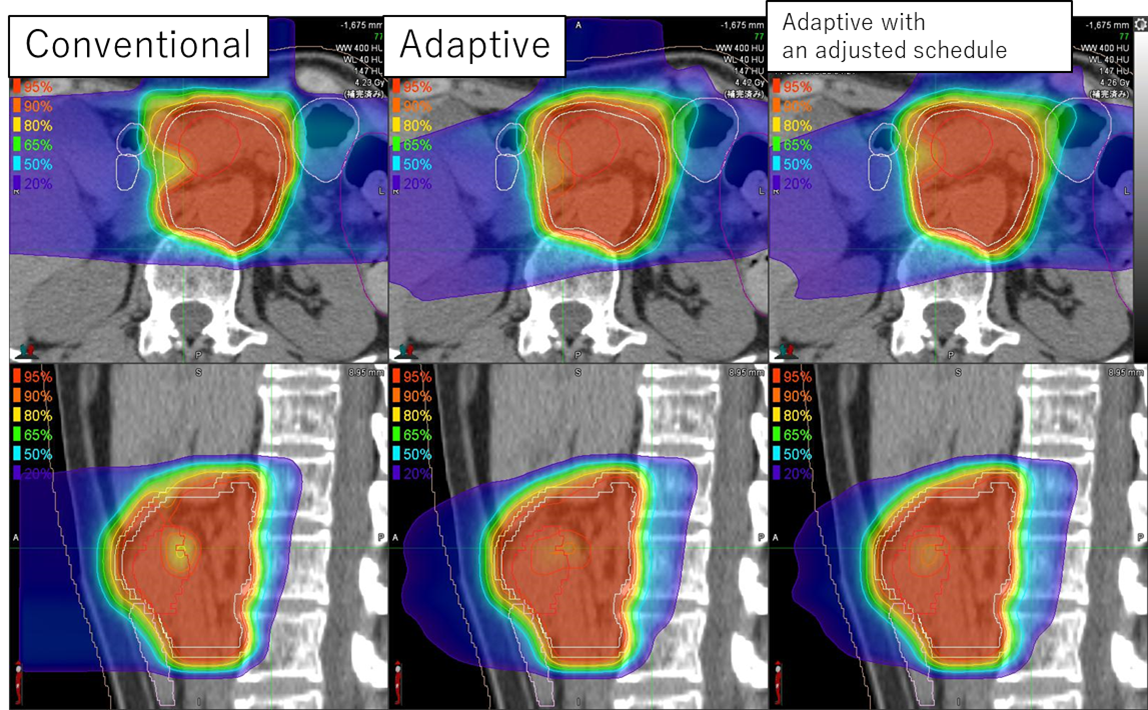
Figure S3. Dose distributions in the axial and sagittal planes of the accumulated dose. Red, white, and cream lines indicate the gross tumor volume, clinical target volume, and planning target volume, respectively.
